# The Effects of Tryptamine Psychedelics in the Brain: A meta-Analysis of Functional and Review of Molecular Imaging Studies

**DOI:** 10.3389/fphar.2021.739053

**Published:** 2021-09-29

**Authors:** João Castelhano, Gisela Lima, Marta Teixeira, Carla Soares, Marta Pais, Miguel Castelo-Branco

**Affiliations:** CIBIT/ ICNAS, Faculty of Medicine, University of Coimbra, Coimbra, Portugal

**Keywords:** psychedelic agents, functional magnetic resonance imaging, positron emission tomography, cognition, 5-hydroxytryptamine receptor 1A, 5-hydroxytryptamine receptor 2A, serotonin

## Abstract

There is an increasing interest in the neural effects of psychoactive drugs, in particular tryptamine psychedelics, which has been incremented by the proposal that they have potential therapeutic benefits, based on their molecular mimicry of serotonin. It is widely believed that they act mainly through 5HT2A receptors but their effects on neural activation of distinct brain systems are not fully understood. We performed a quantitative meta-analysis of brain imaging studies to investigate the effects of substances within this class (e.g., LSD, Psilocybin, DMT, Ayahuasca) in the brain from a molecular and functional point of view. We investigated the question whether the changes in activation patterns and connectivity map into regions with larger 5HT1A/5HT2A receptor binding, as expected from indolaemine hallucinogens (in spite of the often reported emphasis only on 5HT2AR). We did indeed find that regions with changed connectivity and/or activation patterns match regions with high density of 5HT2A receptors, namely visual BA19, visual fusiform regions in BA37, dorsal anterior and posterior cingulate cortex, medial prefrontal cortex, and regions involved in theory of mind such as the surpramarginal gyrus, and temporal cortex (rich in 5HT1A receptors). However, we also found relevant patterns in other brain regions such as dorsolateral prefrontal cortex. Moreover, many of the above-mentioned regions also have a significant density of both 5HT1A/5HT2A receptors, and available PET studies on the effects of psychedelics on receptor occupancy are still quite scarce, precluding a metanalytic approach. Finally, we found a robust neuromodulatory effect in the right amygdala. In sum, the available evidence points towards strong neuromodulatory effects of tryptamine psychedelics in key brain regions involved in mental imagery, theory of mind and affective regulation, pointing to potential therapeutic applications of this class of substances.

## Introduction

Pharmacologic challenges with tryptamine hallucinogen substances have been used as models for psychosis. In recent years, many studies have used substances to study the neuronal correlates of altered states of consciousness (dos Santos et al., 2016). A current research trend involves testing the effects of hallucinogens as potential therapeutic alternatives for psychiatric disorders (Kraehenmann, 2017; Lowe et al., 2021). Here we aimed to perform a quantitative meta-analysis of neuroimaging studies in this field. The current work summarizes the level of (in) consistency between functional imaging outcomes from connectivity and activation studies that might help to further clarify the implication of previous reports and their importance concerning the therapeutic potential of these drugs.

The relation between psychedelic experience and psychosis remains intriguing (Cumming et al., 2021). Sensory hallucinations and attentional deficits are common manifestations in schizophrenia and other neuropsychiatric disorders. The neural correlates of visual and auditory alertness in these conditions have been a matter of study. The approach of experimentally inducing states of psychosis was proven to be very useful to understand the effects of distinct substances in the brain in the so–called pharmacological fMRI approach (Daumann et al., 2010). In particular, neuroimaging studies have investigated the neural correlates of alertness based on agonistic modulation of the human serotonin 2A receptor (5-HT2AR, 5-hydroxytryptamine2A) (using dimethyltryptamine-DMT) and N-methyl-D-aspartic acid (NMDA) antagonism (using ketamine) for psychosis (Daumann et al., 2010). Moreover, 5-HT2AR activation through LSD has been implicated in the formation of visual hallucinations and cognitive impairments (Schmidt et al., 2018). The psychedelic experience produced by psilocybin (Psi) (a substance found in “magic mushrooms”) is characterized by “unconstrained” cognition and profound alterations in the perception of time, space and selfhood (Mason et al., 2021). This substance is a preferential serotonin (5-HT) 2A/1A receptor agonist (Halberstadt and Geyer, 2011). Psilocybin, reduces the processing of negative stimuli (Preller et al., 2017) which is relevant concerning affective processing in the amygdala. This emotion-processing structure is particularly prone to serotonergic modulation. Psilocybin-induced decrease in amygdala reactivity correlates with and reduces threat-induced modulation of amygdala activation and/or connectivity (Kraehenmann et al., 2015, Kraehenmann et al., 2016; Preller et al., 2017; Barrett et al., 2020b).

Other hallucinogens inducing similar effects have been used to study the rapid changes in brain dynamics and functional connectivity (FC) in neuroimaging, regarding the quality of conscious experience in the psychedelic state (Tagliazucchi et al., 2014, Tagliazucchi et al., 2016; Luppi et al., 2021). These substances include Lysergic acid diethylamide (LSD) that induces profound changes across various mental domains, including perception, self-awareness and emotional state (Mueller et al., 2017; Luppi et al., 2021); or Ayahuasca, that is a beverage traditionally used by Amazonian Amerindians composed by a mixture of compounds that increase monoaminergic transmission. Ayahuasca caused significant decreases in the activity and connectivity of the default mode network (DMN) (Palhano-Fontes et al., 2015) and increased excitability in multimodal brain areas as the posterior association cortex, the cingulate, and the Medial temporal lobe (MTL) (Riba et al., 2004, Riba et al., 2006), that are pivotal in interoception and emotional processing.

Psychedelic drugs such as LSD were used extensively in psychiatry in the past and their therapeutic potential is beginning to be re-examined today (Kaelen et al., 2016; Kraehenmann, 2017). Accordingly, the use of these substances may have important implications for the treatment of depression, mood and anxiety disorders (Kraehenmann et al., 2015). Additionally, the current literature also emphasizes the importance of 5-HT2A/1A receptor subtypes in the control of social functioning, and as prospective targets in the treatment of sociocognitive impairments in psychiatric illnesses (Preller et al., 2016). Here we provide a comprehensive review of studies in this field. Our findings suggest important implications for the understanding of the mechanism of action of hallucinogenic drugs and provide further insight into the role of these substances to improve mental health, pain or neurodegenerative disorders.

## Methods

### Search Strategy and Data Sources

**FIGURE 1:**
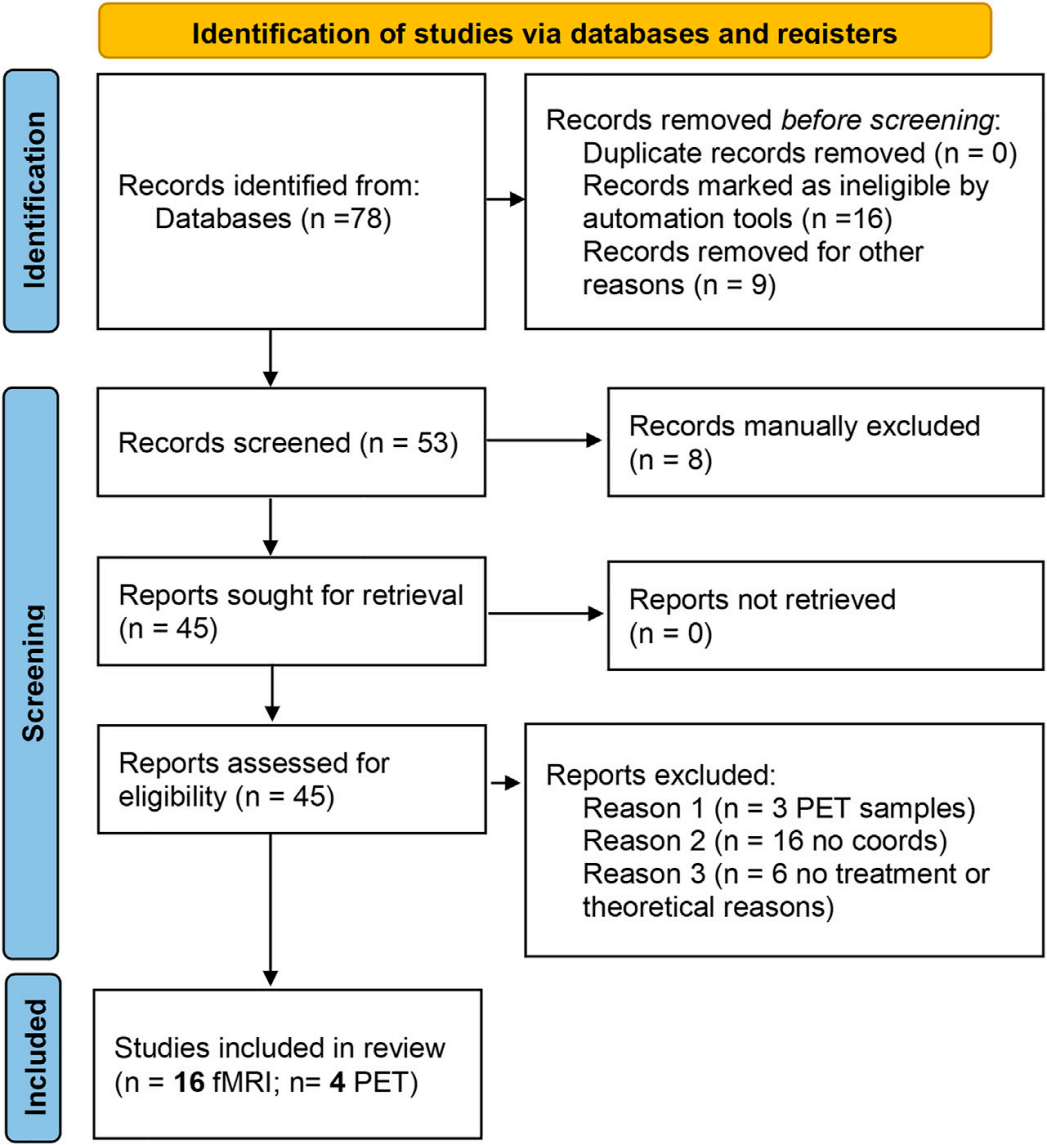
PRISMA flow diagram for the meta-analysis. The summary of papers identified through databases search, screened for the inclusion/exclusion criteria and included in the final analysis are reported in the standard PRISMA diagram.

We performed the literature search using the PubMed database in Sep/2020. The search criteria were: LSD (Title/Abstract) OR lysergic (Title/Abstract) OR psilocybin (Title/Abstract) OR ayahuasca (Title/Abstract) OR dimethyltryptamine (Title/Abstract) AND [fMRI (Title/Abstract) OR BOLD (Title/Abstract) OR PET (Title/Abstract)]. Figure 1 (PRISMA) summarizes the number of articles and duplicates that were found. To identify functional brain imaging studies, our inclusion criteria were: 1) the studies imaged the whole brain; 2) the results presented coordinate-based data in a standard space and were not review papers; 3) the imaging method was fMRI or PET; 4) subjects were healthy controls; 5) sample size N ≥ 8 (Eickhoff et al., 2016).

From the initial identification of 78 studies, the final study included 16 fMRI studies and four PET studies (Table 1) reporting brain imaging experiments related to those drugs. We then used the foci of brain activations extracted from each of the included studies for the ALE analysis.

**TABLE 1:** **Summary of studies included in the review**. The studies detail and individual results are reported.

**MRI Studies**	**N**	**Age**	**Drug**	**Task**	**Signal**	**Result**	**Magnet (T)**
Daumann J, 2008	14	26–42	DMT	Covert orienting of attention task/Button press	Bold	Administration of Sketamine, yet not DMT, yielded a stronger signal increase in cortical regions involved in the modulation of inhibition of return	1.5
Dauman J, 2010	14	26–42	DMT	Visual and the auditory target detection/button press	Bold	DMTdecreased bold response for visual task, particularly in extrastriate regions during auditory in temporal regions. S-ketamine led to increased cortical activation in the left insula and precentral gyrus in the auditory modality	1.5
Kraehenmann R, 2014	25	21–27	Psi	Emotion picture discrimination	Bold	Decrease in Amygdala reactivity	—
Tagliazucchi E, 2014	15	23–41	Psi	Resting	Bold and power	Increased cortical BOLD variance and total spectral power	3
Kaelen M, 2016	12	21-	LSD	Resting state and music listen	Connect	Increased PHC–visual cortex functional connectivity	3
Palhano-Fontes FM, 2015	10	24–48	Aya	Verbal fluency task and RS	Connect	Connectivity within the PCC/Precuneus decreased. Modulation of the activity and the connectivity of the DMN	1.5
Kraehenmann R, 2016	25	21–28	Psi	Emotional (threat and neutral) picture discrimination	Connect	Reduces threat-induced modulation of amygdala connectivity to primary visual cortex	3
Preller K, 2016	21	20–37	Psi	Cyberball-social exclusion game	Connect	Neural response to social exclusion was decreased in the dorsal anterior cingulate cortex (dACC) and the middle frontal gyrus. Psi reduced the perception of social pain	3
Mueller F, 2017	20	25–58	LSD	Gender discrimination task	Bold	Significant effect of LSD on the left amygdala	3
Muller F, 2017	20	25–60	LSD	Resting state	Connect	Increased thalamic resting-state connectivity	3
Peller A, 2017	22	20–34	LSD	Music paradigm	Bold	Increased signal in the left SMA. LSD increased the attribution of meaning to previously meaningless music	3
Schmidt A, 2017	18	25–58	LSD	Go-no go task	Bold	LSD administration impaired inhibitory performance and reduced brain activation	3
Muller F, 2018	20	25–60	LSD	Resting state	Connect	LSD administration significantly decreased functional connectivity within visual, sensorimotor and auditory networks and the default mode network	3
Preller K, 2018	24	20–34	LSD	Social interaction task	Bold	LSD reduced activity in brain areas important for self-processing and social cognition	3
Preller K, 2019	25	20–34	LSD	Resting state	Connect	LSD increased effective connectivity from the thalamus to the posterior cingulate cortex	3
Smigielski L, 2019	38	40–60	Psi	Resting state (RS), focused attention (FA), and open awareness (OA) meditation	Connect	Long lasting alterations in anterior–posterior DMN	3
** PET studies**	**N**	**Age Range**	**Drug**	**PET scan**	—	**Results**	—
Vollenweider F 1997	10	26–43	Psi	FDG	—	Cerebral metabolic rate of glucose (CMRglu) increases in the frontomedial and frontolateral cortex, anterior cingulate and temporomedial cortex	—
Gouzoulis-Mayfrank E 1999	32	27–47	Psi and Methamphetamine	FDG	—	MDE and METH induced cortical hypometabolism and cerebellar hypermetabolism. In the MDE group, cortical hypometabolism was more pronounced in frontal regions, with the exception of the right anterior cingulate	—
Vollenweider F 1999	7	25–30	Psi	(11C) raclopride D2 -dopamine receptors	—	Psilocybin significantly decreased [11 C]raclopride receptor binding potential (BP) bilaterally in the caudate nucleus and putamen	—
Madsen M 2019	8	26–40	Psi	5-HT2AR agonist radioligand (11C) Cimbi-36	—	Intake of psilocybin leads to significantly 5-HT2AR reduced occupancy in the human brain	—

### Data Extraction

We exported foci data manually from each paper to a text file containing all the coordinates of the results from the original studies that passed the inclusion criteria. All coordinates were converted to MNI standard space (using the Brett transform as implemented in the tal2mni/mni2tal function of MATLAB (R2020a, Mathworks, United States). It is important to note that all MRI studies included placebo (control) groups and the data reported are comparisons of drug vs placebo effects.

Additionally, all the available PET studies (with different tracers) are discussed in a narrative manner, given the insight they provide on molecular mechanisms of action.

### ALE Analysis

ALE meta-analysis was carried out as described previously by (Turkeltaub et al., 2002). To assess the statistical significance of the results we used a permutation test (1,000 permutations) and set a threshold *p* value < 0.001 and a minimum cluster size of 200 mm^3^ (Eickhoff et al., 2016). We used GingerALE (v3.0.2), the Java version of ALE developed at the Research Imaging center and available at http://brainmap.org/ale for data processing. For visualization, the results were overlaid into a standard MNI image template (Kochunov et al., 2002).

Since we found fMRI experiments with BOLD and connectivity results, we performed an ALE including all fMRI papers and two other separate analysis: 1) using the results from the BOLD amplitude changes; 2) using the connectivity results from the fMRI papers. The resulting ALE images were converted to Z scores in order to simplify interpretation and show their significance.

Activation maps related to each of the tasks were overlaid and displayed using Mango software (http://ric.uthscsa.edu/mango/) and the Talairach Daemon (http://talairach.org/) tool was used to extract anatomical labels of results. All the input files used in our analysis and output results are freely available upon request to the corresponding author.

## Results

Published papers were screened for the methodological information. A total of 78 papers were initially included (Figure 1 summarizes the number of papers and number of excluded at each stage as a flow PRISMA diagram; see Table 1). All included studies have N > 8 subjects (range 10–38 participants; Median = 20, total of 323 participants for fMRI studies and 57 for PET studies). These studies included BOLD, Connectivity and PET studies. Table 1 reports the demographic information of the selected datasets, the drugs in use, experimental task and a descriptive summary of the individual results. Detailed information about the design, doses, route of administration and comparators are presented in Supplementary Table S1.

A total of 323 subjects participated in this set of Psychoactive studies that include LSD, Psilocybin, Ayahuasca and DMT. The age range of the participants was 20–60 years. In total, there were 98 foci for the BOLD studies and 76 foci in the connectivity studies that were included in the meta-analysis.

We performed quantitative ALE meta-analysis using fMRI activation data both for BOLD and connectivity reports. The individual meta-analysis of brain activation and connectivity associated with psychoactive drugs revealed eleven clusters of reliable activation and connectivity modulation across studies. Table 2 identifies the coordinates of the peak voxel of each cluster and the brain region label including statistical values. There, MNI coordinates and the ALE values of the clusters are reported. We found a set of areas that are affected by psychoactive drugs and those areas are mainly located at frontal, parietal and limbic lobes. In particular, Putamen and Anterior cingulate activations are reported with highly significant alterations (*p* < 0.00001).

**TABLE 2:** **Overlap in brain activation across studies**, as assessed using a quantitative meta-analysis of BOLD and connectivity studies. The major activations are shown with their corresponding Brodmann Area (BA), the ALE value of the peak activated voxel and MNI coordinates. Statistical values are also reported for each cluster.

**fMRI (BOLD + Connect.)**										
**Cluster #**	**x**	**y**	**Z**	**ALE**	**P**	**Z**	**Hemis**	**Lobe**	**Label**	**BA**
1	26	0	−14	0.0178	0.00000	4.62	R	Sub-lobar	Lentiform Nucleus	Putamen
2	6	24	18	0.0135	0.00006	3.83	R	Limbic Lobe	Anterior Cingulate	33
3	−2	−46	30	0.0159	0.00001	4.32	L	Limbic Lobe	Cingulate Gyrus	31
4	54	32	20	0.0158	0.00001	4.29	R	Frontal Lobe	Middle Frontal Gyrus	46
5	48	−66	26	0.0147	0.00002	4.09	R	Temporal Lobe	Middle Temporal Gyrus	39
6	−6	44	−4	0.0147	0.00002	4.08	L	Limbic Lobe	Anterior Cingulate	32
7	−52	−46	36	0.0160	0.00001	4.32	L	Parietal Lobe	Supramarginal Gyrus	40
8	50	−68	−2	0.0138	0.00005	3.89	R	Occipital Lobe	Inferior Temporal Gyrus	37
9	48	46	6	0.0135	0.00006	3.84	R	Frontal Lobe	Middle Frontal Gyrus	10
10	−38	−68	−18	0.0134	0.00007	3.82	L	Posterior Lobe	Declive	*
11	−42	40	24	0.0129	0.00010	3.72	L	Frontal Lobe	Superior Frontal Gyrus	9
**BOLD**										
**Cluster #**	**X**	**Y**	**Z**	**ALE**	**P**	**Z**	**Hemis**	**Lobe**	**Label**	**BA**
1	48	−66	26	0.0147	3.6E-06	4.49	R	Temporal Lobe	Middle Temporal Gyrus	39
2	50	−68	−2	0.0138	1.0E-05	4.26	R	Occipital Lobe	Inferior Temporal Gyrus	37
3	26	−2	−16	0.0099	2.0E-04	3.54	R	Limbic Lobe	Parahippocampal Gyrus	Amygdala
3	24	−4	−22	0.0090	3.6E-04	3.38	R	Limbic Lobe	Parahippocampal Gyrus	Amygdala
4	−40	−80	−8	0.0110	9.6E-05	3.73	L	Occipital Lobe	Fusiform Gyrus	19
**Connectivity**										
**Cluster #**	**X**	**y**	**Z**	**ALE**	**P**	**Z**	**Hemis**	**Lobe**	**Label**	**BA**
1	−38	−68	−18	0.0134	7.1E-06	4.34	L	Posterior Lobe	Declive	*
2	10	-68	22	0.0128	1.2E-05	4.22	R	Limbic Lobe	Posterior Cingulate	31
3	0	48	−12	0.0132	8.4E-06	4.30	L	Limbic Lobe	Anterior Cingulate	32
4	−48	−74	2	0.0121	2.0E-05	4.10	L	Occipital Lobe	Inferior Temporal Gyrus	*

**FIGURE 2:**
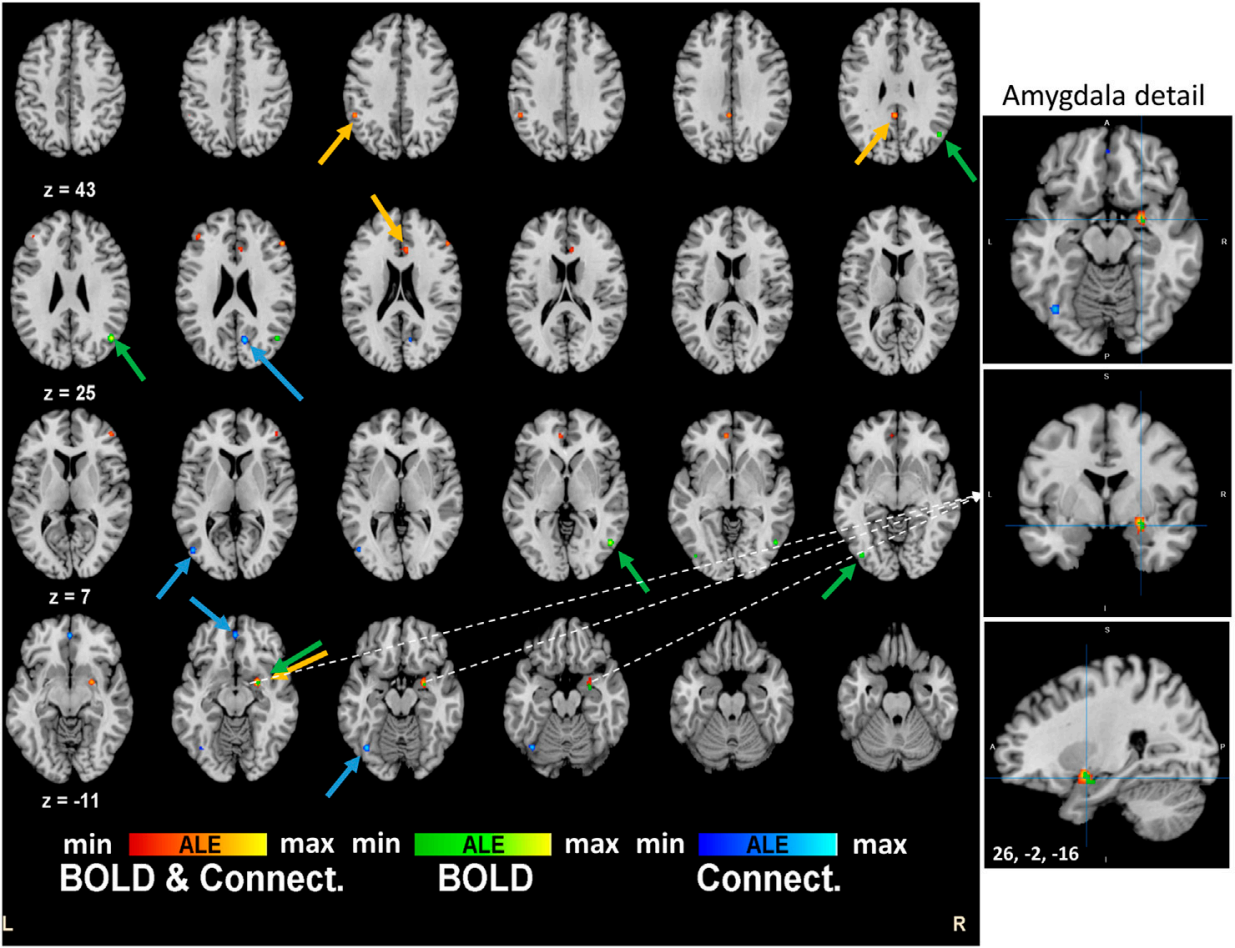
Brain activation maps for tryptamine psychedelics studies. An extended network shows up in the quantitative meta-analysis. Frontal decision related areas and other visuo-temporal areas are affected by the drug. Particularly, right amygdala is implicated in the effects of the psychedelics drugs. These results are significant at *p* < 0.0001.

Regarding separate BOLD and connectivity results (Table 2), the ALE analysis shows reliable alterations (mainly deactivations) that strongly appear in the Amygdala, temporal gyrus and fusiform gyrus (*p* < 0.0004; Zmin = 3.38) for the BOLD studies when participants receive the psychoactive drugs irrespectively of the task in hand and mainly at the right hemisphere (Figure 2). On the other hand, connectivity studies analysis revealed a distributed network of changed connections in the left hemisphere when participants are under the effect of psychoactive drugs. This network includes particularly the cingulate cortex (Brodmann areas 31 and 32; *p* < 0.000013; Z = 4.22) and the inferior temporal gyrus (*p* < 0.00002; Z = 4.10) in the occipital lobe. Figure 2 depicts the brain maps of concordant clusters of significant alterations (p < 5E-4) during psychoactive drug experiments.

Additionally, we performed a systematic review of the PET studies in the field. Surprisingly, we only found four studies that passed the inclusion criteria. These studies reported results for distinct PET tracers namely (18F) DG and (11C) Cimbi-36, only the latter being related to 5-HT2AR, a serotonin receptor for which there is wide evidence for psychoactive drug effects. Other studies have addressed the distribution of 5-HT2A receptors such (18F) altanserin (11C) Cimbi-36 or (18F) setoperone but with no direct link to the effects of hallucinogens. For example, the PET study from Stenbæk et al., 2018 in 159 participants shows that differences in 5-HT2AR availability are not related to variations in trait Openness in healthy individuals, which is at odds with the notion that putative stimulation of the 5-HT2AR with compounds such as psilocybin may contribute to long-term changes in trait Openness. This study, which was not formally included because psilocybin or other hallucinogens were not administered, shows that in any case there is no evidence in favor of an association between 5-HT2AR and trait Openness, ruling out a simple link between this trait and 5-HT2AR effects of psylocibin.

This concept that neural effects stem mainly from 5-HT2AR has been challenged for indoleamine/tryptamine hallucinogens. A large body of evidence demonstrates indeed that both 5-HT1A and 5-HT2A receptors are responsible for the behavioral effects of these hallucinogens (Halberstadt and Geyer, 2011). These authors point out that, in general, different neurotransmitter systems contribute to the effects of indoleamine/tryptamine hallucinogens, which in the case of LSD involves also dopamine receptors.

Contrary to the MRI studies that report effects of using several distinct hallucinogenic drugs, the PET studies focused on the effects of the Psilocybin. These molecular studies reveal distinct 5-HT2AR receptor occupancy and density as a consequence of Psilocybin intake (Madsen et al., 2021). There was a decrease in receptor binding particularly in frontal regions. While this confirms the action of Psilocybin at the level of these receptors it does not preclude actions in other neurotransmitter systems. Accordingly, Psilocybin significantly decreased [11 C]raclopride receptor binding potential (BP) bilaterally in the caudate nucleus and putamen (Vollenweider et al., 1999) showing that effects are not at all exclusive to the 5-HT2AR system, but include the D2 dopamine receptor.

Concerning 18-FDG studies, Vollenweider et al. (1997) suggested that Psilocybin induced “metabolic hyperfrontaly”, as encountered in baseline states of psychosis. Using the same radiotracer, Gouzoulis-Mayfrank et al. (1999) partially replicated these findings by showing that psilocybin increased metabolism in distinct right hemispheric frontotemporal cortical regions, particularly in the anterior cingulate, in contrast with the thalamus. More placebo controlled molecular imaging studies are needed to understand the impact of tryptamine hallucinogens in the brain. Nevertheless, molecular imaging atlas of different 5-HT receptor systems (Beliveau et al., 2017) suggest that the regions found in most fMRI studies share a sizable density of both 5-HT1A and 5-HT2A receptors.

## Discussion

### Psychological Effects

**FIGURE 3:**
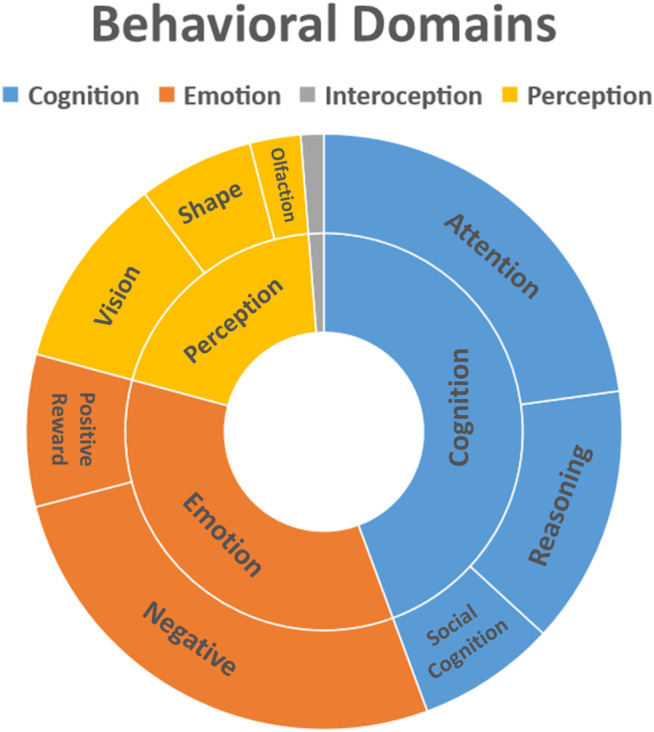
Behavioral domains affected by use of psychoactive substances. Behavioral data was extracted from Mango plugin (Behavioral Analysis Plugin v3.1) for the clusters obtained from the quantitative ALE analysis (Z-score>2.39).

The profound experience induced by psychedelics like DMT, Ayahuasca, LSD and Psilocybin is characterized by changes in emotion, perception and cognition, visual imagery and differences in the sense of self (Swanson, 2018; Barrett et al., 2020b; Lowe et al., 2021; Luppi et al., 2021). Figure 3 summarizes these effects.

### Amygdala and Emotional Effects and Anxiety

The most obvious finding of our analysis is the deactivation of the amygdala during the psychedelic induced states, which might underlie the emotional effects of these substances. Altered processing of facial expressions with negative valence and modulation of the amygdala activity to these stimuli has been found after the administration of serotonergic psychedelics on healthy and clinical populations (Rocha et al., 2019). The decreased reactivity of the amygdala to negative stimuli was also associated with an increase of positive mood states during the acute phase (Kraehenmann et al., 2015) and also long-term (Barrett et al., 2020). These effects may be of clinical relevance in disorders associated with difficulties in emotional processing such as depression, anxiety and addiction. Previous studies evaluating anxiety disorders have found consistent findings on the role of the amygdala in the symptoms of fear and anxiety (Holzschneider and Mulert, 2011). Meta-analytic evidence revealed consistent hyperactivation of the amygdala in post-traumatic stress disorder, social anxiety disorder and specific phobia, as well as during fear conditioning in healthy subjects, suggesting a common excessive engagement of fear circuitry (Etkin and Wager, 2007). Our results also show a greater deactivation in the right amygdala. Although there are no conclusive findings on the lateralization of amygdala in emotional processing (Kraehenmann et al., 2015), some studies point to different activations. During the presentation of emotional stimulus, right amygdala hyperactivation was observed in patients with PTSD compared with trauma-exposed non-PTSD individuals (Brohawn et al., 2010), as well as in patients with obsessive-compulsive disorder compared with healthy controls (Thorsen et al., 2018). In the latter, right amygdala hyperactivation was more evident in unmedicated patients. An increased influence from right amygdala to right middle frontal gyrus and a decreased influence from right precuneus to right amygdala was also associated to the trait neuroticism, which is the tendency to experience negative emotional states and negative self-referential information processing (Pang et al., 2016). Further research should be undertaken to elucidate the long-term impact of psychedelics on amygdala responsiveness. Recent findings from healthy populations indicated a reduced amygdala response to facial stimuli 1-week post-psilocybin, returning to baseline after 1 month (Barrett et al., 2020). Nevertheless, an increased reactivity was found in clinical populations 1 day after psilocybin session (Roseman et al., 2018).

### Salience Network and Pain, Psychiatric and Neurological Disorders

Another important finding was the deactivation of brain areas associated with the Salience Network (SN), such as the dorsal anterior cingulate cortex. This network is involved in attributing salience and selecting relevant interoceptive, autonomic and emotional stimuli (Menon, 2015). Dysfunctions on salience-processing are relevant in many psychiatric and neurological disorders, such as schizophrenia, dementia, autism, mood and anxiety disorders, drug addiction and pain (Menon, 2015; Uddin, 2015). An aberrant salience attribution to internal stimuli is proposed as a model for psychosis (Kapur, 2003), and is also conceptualized as having an important role in the symptoms of delusions and hallucinations in schizophrenia (Palaniyappan and Liddle, 2012). These findings may help to understand the early research on psychedelics as models for psychosis. A salience network dysfunction hypothesis is also considered in autism spectrum disorder, which suggests that impaired attribution to sensory stimuli might be associated with dysfunctional cognitive processes, such as social cognition (Uddin, 2015).

### Theory of Mind and Social Cognition

We also found relevant patterns in regions involved in theory of mind such as supramarginal gyrus, medial prefrontal cortex, precuneus and posterior cingulate cortex. LSD decreased the efficiency of establishing joint attention in the PCC and the temporal gyrus, an effect attributed to 5-HT2AR stimulation (Preller et al., 2018). The authors suggested a decreased differentiation between the self and the other during social interactions. This altered sense of self characterized by a decreased differentiation between self-representations and other-representations is usually called “ego dissolution” (Nour et al., 2016). In addition, psilocybin decreased the feeling of social exclusion processing in the ACC (Preller et al., 2016). These findings point to the modulation of social cognition, which may be an important mechanism contributing to the therapeutic potential of psychedelics (Preller and Vollenweider, 2019). There is evidence for the role of the supramarginal gyrus, highlighted in our analysis, in overcoming emotional egocentricity bias in social judgements (Silani et al., 2013), which suggests a possible role in empathy. The overlap between some areas involved in theory of mind and the default mode network (DMN) has led some authors to suggest the role of the DMN in the social understanding of others (Li et al., 2014), as well as the role of the PCC in attributing mental states to others (Mars et al., 2012). Tagliazucchi et al. (2016) reported that LSD-induced states increased functional connectivity in bilateral temporo-parietal junction, a key component of theory of mind, which was correlated to subjective reports of ego dissolution. In line with this, previous studies indicated the effects of tryptamine psychedelics on dimensions related to healthy social functioning such as increased emotional empathy and prosocial behaviour (Dolder et al., 2016; Pokorny et al., 2017; Mason et al., 2019; Uthaug et al., 2021), changes in personality traits agreeableness (Netzband et al., 2020) and compassion (Apud Peláez, 2020), as well as feelings of connection to others (Watts et al., 2017). A recent study in mice reported that repeated administration of low doses of LSD promoted social behaviour by potentiating 5-HT2AR and AMPA receptor neurotransmission in the mPFC via an increasing phosphorylation of the mTORC1 (de Gregorio et al., 2021).

### Mental Imagery

The activation of visual areas by psychedelics induced substances, was another outcome of our quantitative meta-analysis, namely visual areas BA19 and visual fusiform region BA37. These areas are densely populated with 5-HT2A receptors. Various studies indicated a key function for 5-HT2ARs in visual processes and the pathogenesis of visual hallucinations (Moreau et al., 2010; Seillier et al., 2017). Classical hallucinogens are used as models for studying the pathophysiology of different neuropsychiatric conditions with positive psychotic symptoms, such as schizophrenia, Parkinson’s and Alzheimer’s disease, which alter individual visual and perceptual experiences. The activation of 5-HT2ARs increases the excitability of the visual cortex in the absence of external visual stimulation (Moreau et al., 2010). In addition, the activation of 5-HT2ARs mediates the visual hallucinations that are generated by serotonergic hallucinogens, such as LSD or psilocybin (Nichols, 2004; Vollenweider and Kometer, 2010). In line with this, the hallucinogen-induced decrease in alpha oscillations might allow spontaneous self-organized activity to gain perceptual quality (Kometer et al., 2013). Recent studies demonstrated that acute LSD administration to healthy subjects not only produces elementary and complex visual (pseudo)hallucinations and perceptual illusions (Carhart-Harris et al., 2016b; Preller et al., 2017; Schmid et al., 2015; Schmidt et al., 2018), but also impaired inhibitory processes (Schmid et al., 2015) and cognitive organization (Carhart-Harris et al., 2016a). Impairments in inhibition after psilocybin administration and cognitive impairments after LSD administration were attenuated by administration of the 5-HT2AR antagonist ketanserin (Quednow et al., 2012; Preller et al., 2017). However, this does not exclude the contribution of other receptor subtypes such as 5-HT1AR (Halberstadt and Geyer, 2011). 5-HT2AR activation is indeed pivotal in inducing visual hallucinations but other receptors also contribute to cognitive impairments, and their abnormal activity can be associated with cognitive deficits in neuropsychiatric disorders such as schizophrenia and Alzheimer’s disease (Švob Štrac et al. 2016). Schmidt et al. (2018) proposes that psychedelics disrupt information processing in inhibitory cortico-striato-thalamocortical (CSTC) feedback loops that have been implicated in sensory gating of internal and external information to the cortex. This psychedelic-induced disinhibition might lead to an inability to filter and inhibit exteroceptive and interoceptive stimuli, resulting in high-level processing overload and the formation of hallucinations.

In line with our results, De Araujo et al. (2012) investigated the neuronal mechanisms underlying psychedelic-induced visual mental imagery using functional magnetic resonance imaging (fMRI). The authors found that ayahuasca increased activations in mental imagery networks, including early visual areas (BA 17, 18, 19), parahippocampal gyrus, middle temporal cortex, and frontal cortex (BA10). They also showed that ayahuasca-induced changes in primary visual cortex (BA17) were preceding activation patterns in higher-level areas, indicating that ayahuasca-induced imagery is initiated in BA17, but activity is spread to higher-level cortical areas involved with episodic memory retrieval and the processing of contextual associations, such as BA30 and BA37, which might feed memory-related content. In addition to perceptual alterations of simple and elementary visual features as color, brightness, visual contrast (Klüver, 1942; Rummele and Gnirss, 1961; Kometer et al., 2013; Kometer and Vollenweider, 2018) that might be explained by increased excitation in V1 (Kometer et al., 2013; Császár-Nagy et al., 2019), complex imagery and hallucinations has been reported (Császár-Nagy et al., 2019; Díaz, 2010; dos Santos et al., 2016; Kraehenmann, 2017; Kometer et al., 2013), with personal and profound significance, stemming from autobiographical memory (Studerus et al., 2011) to current life situations (Shanon, 2010) charged with emotional content. These complex forms of hallucinogen-induced hallucination and visions, also lead the recruitment of higher level regions in the brain, given that psychedelic imagery is usually very structured, thematic and personal (Kraehenmann, 2017). In accordance to our main results, studies have reported visual hallucinations caused by neuronal stimulation of PFC (Blanke et al., 2000), temporal areas (Mégevand et al., 2014; Aminoff et al., 2016) and increased functional connectivity between PFC and primary visual cortex (Carhart-Harris et al., 2016b). Furthermore, the review conducted by dos Santos et al. (2016), suggested that hallucinogens increase introspection and positive mood by modulating brain activity in the fronto-temporo-parieto-occipital cortices. Neuromodulatory changes induced by tryptamine psychedelics can give significant input to the study of neuropsychiatric conditions where similar patterns of activation or connectivity (Barrett et al., 2020b; Madsen et al., 2021) are found and to the implementation of new pharmacological or psychotherapeutic interventions taking advantage of this link between visual imagery, autobiographical memory and emotions (Barrett et al., 2020).

The role of amygdala in this interplay is not of less importance. The amygdala plays an important role in emotional visual processing (Vuilleumier et al., 2004). Important networks between amygdala and ventral visual pathways in primates are reported (Freese and Amaral, 2005), as well as the role of the amygdala in visual awareness (Duncan and Barrett, 2007). Furl et al., 2013 suggested that the amygdala modulates visual processing by feedback connections and that it may have a contextual role during visual coding. Deactivation of the amygdala during the psychedelic induced states has been consistently found and along with the inhibition of DMN opens a therapeutic potential for accessing and transforming autobiographical memories, emotions and maladaptive perceptions.

### Default Mode Network

In addition to the mechanisms described above, changes in Default Mode Network (DMN) connectivity may be another neural basis involved in the psychologic and therapeutic effects attributed to tryptamine psychedelics. DMN areas present lower levels of activity when individuals are engaged in a task requiring externally oriented attention and activate during passive rest states or internally oriented mental processes, such as autobiographical memory, mind wandering, self-reflective thought, and future thinking (Buckner et al., 2008; Andrews-Hanna et al., 2010).

In our analysis, a decreased connectivity within PCC/Precuneus, key components of the DMN, was observed. Regarding classic hallucinogens, studies revealed that psilocybin, LSD, and ayahuasca could decrease DMN functional integrity (Carhart-Harris et al., 2012, Carhart-Harris et al., 2016c; Palhano-Fontes et al., 2015; Luppi et al., 2021; Madsen et al., 2021; Mason et al., 2021),. Barrett et al. (2020b), recently proposed that Psilocybin alters default mode network integrity and fronto-parietal network modularity by reducing Claustrum functional connectivity with these circuits. This study showed that psilocybin reduced activity of left and right claustrum during the acute effects of psilocybin, leading to alterations in claustrum connectivity with brain networks that support both sensory and high-level cognitive processes. Specifically, the authors found decreased connectivity between claustrum and the DMN during the effects of psilocybin, decreased connectivity between left claustrum and fronto-parietal task control circuits and increased connectivity between right claustrum and the same fronto-parietal networks. In sum they assigned to the claustrum (dense in 5-HT_2A_ receptors ) a role in the psilocybin-induced disruption in both the DMN and task-positive networks. Accordingly, Madsen et al. (2021) found negative correlations between the DMN integrity and the plasma psilocin levels and subjective drug intensity. These results support the proposed theory of action for psychedelics to decrease the control of top-down structures and increase the excitability of areas involved in sensory, emotional and cognitive appraisal processes. (Barrett et al., 2020b; Mason et al., 2021). The expression and awareness of normally repressed information would explain the novelty of the experience and the new associations would facilitate the formation of new insights (Domínguez-Clavé et al., 2016; McKenna and Riba, 2018). In line with this, Mason et al. (2021) reported psilocybin-induced decreased within-network connectivity of the DMN and increased functional connectivity between the DMN and the Frontoparietal Network (FPN) and between the DMN and the Salience Network (SN), which predicted higher scores in aspects of creative thinking and long-term increases in novelty of generated ideas. However, in contrast to these findings, there have also been findings of increased DMN activity by hallucinogens (Carhart-Harris et al., 2017; Kometer et al., 2015; Petri et al., 2014; Tagliazucchi et al., 2014). Regarding the associated therapeutic potential, DMN activity is increased in depression (Sheline et al., 2009) acute and chronic pain (Alshelh et al., 2018), schizophrenia (Garrity et al., 2007) and Parkinson’s disease (Van Eimeren et al., 2009) Aberrant patterns of connectivity are also found in drug addiction (Zhang and Volkow, 2019) and eating disorders (Stopyra et al., 2019). It seems to be reduced in autism and in Alzheimer’s disease (Broyd et al., 2009).

### Linking Molecular Imaging and Functional Magnetic Resonance Imaging Data

There were surprisingly few eligible pharmacoimaging studies using PET. Two used FDG (Vollenweider et al., 1997; Gouzoulis-Mayfrank et al., 1999) and together suggested frontal and temporal hypermetabolism, which are consistent with fMRI data. Another used the 5-HT2AR agonist radioligand (11C) Cimbi-36, and showed that intake of psilocybin leads to significantly 5-HT2AR reduced occupancy in the human brain, confirming a role for this receptor subtype. However, a specific link with this receptor system is probably an overstatement, given the evidence that multiple receptors, in particular the 5-HT1AR contribute to the behavioral effects of indoleamine hallucinogens (Halberstadt and Geyer, 2011). The neural effects of these hallucinogens seem to include regions rich in both 5-HT1AR and 5-HT2AR. These probably interact with other receptor systems such as DR2 (Vollenweider et al., 1999), whose binding is decreased probably due to endogenous dopamine release.

### Therapeutic Potential

Taken together, our results support the plausibility of further research on the therapeutic potential of tryptamine psychedelics (Lowe et al., 2021). There is a growing number of clinical trials describing promising data on safety and efficacy of psychedelics and entactogens in several psychiatric disorders, such as posttraumatic stress disorder (Mitchell et al., 2021), treatment-resistant depression (Carhart-Harris et al., 2016a; Palhano-Fontes et al., 2019), substance addictions (Johnson et al., 2014; Bogenschutz et al., 2015); obsessive-compulsive disorder (Moreno et al., 2006); anxiety associated with life-threatening diseases (Gasser et al., 2014; Griffiths et al., 2016; Ross et al., 2016) and social anxiety in autistic adults (Danforth et al., 2018). Those preliminary findings suggest the reduction of depressant, anxiety and addiction symptoms. Patients described feelings of connection, transcendence, insights, self-awareness, alterations in the perception of the self, emotional catharsis, changes in values and life orientations, reconciliations with death, as well as psychological distress (Gasser et al., 2015; Schmid et al., 2015; Belser et al., 2017; Swift et al., 2017; Watts et al., 2017; Noorani et al., 2018; Barone et al., 2019; Lowe et al., 2021), encouraging further studies. Recently, the role of psychedelics in changing behaviours related to healthy lifestyles (Teixeira et al., 2021), as well as a treatment for neurodegenerative disorders (Vann Jones and O’Kelly, 2020) and for pain conditions (Castellanos et al., 2020) has also been hypothesized. Despite the promising results, further work is required to better understand the neurobiological and psychological mechanisms of action and the potential risks underlying the therapeutic action of tryptamine psychedelics. Several questions regarding the long-term impact of psychedelics remain unanswered at the moment. Rigorous research (possibly integrating PET with fMRI (Cumming et al., 2021)) is needed, taking into account the best clinical practices.

## Limitations

A limitation of our analysis is the inclusion of a few studies with relatively small sample sizes, unequal gender distribution and a minority of studies with no control group (this is the case for three PET and two MRI studies). It is nevertheless important to note that all studies included placebo control groups and the data reported are comparisons of drugs vs placebo effects (see Supplementary Table S1). Another caveat is the different substances and doses used, knowing they act on a different range of receptors. The limited number of regions included in the definition of dynamical states in some studies, is also an aspect that should be addressed in future studies.

## Data Availability

The original contributions presented in the study are included in the article/Supplementary Material, further inquiries can be directed to the corresponding author.

## Abbreviations

5-HT1AR, 5-hydroxytryptamine receptor 1A; 5-HT2AR, 5-hydroxytryptamine receptor 2A; ACC, Anterior cingulate cortex; AMPA, α-amino-3-hydroxy-5-methyl-4-isoxazolepropionic acid receptors; BA, Brodmann area; BOLD, Blood oxygenation level dependent; CSTC, Cortico-striato-thalamocortical; DMN, Default mode network; DMT, N,N-Dimethyltryptamine; FC, Functional connectivity; fMRI, Functional magnetic resonance imaging; LSD, Lysergic acid diethylamide; mPFC, Medial Pre-frontal cortex; MTL, Medial temporal lobe; mTORC1, raptor-mTOR protein complex; NDMA, N-methyl-D-aspartic acid; PCC, Posterior cingulate cortex; PET, Positron emission tomography; PFC, Pre-frontal cortex; PTSD, Posttraumatic stress disorder; SN, Salience Network; FPN, Frontoparietal Network.

## References

[B1] Alshelh Z. Marciszewski K. K. Akhter R. Di Pietro F. Mills E. P. Vickers E. R. (2018). Disruption of Default Mode Network Dynamics in Acute and Chronic Pain States. Neuroimage Clin. 17, 222–231. 10.1016/j.nicl.2017.10.019 29159039 PMC5683191

[B2] Aminoff E. M. Li Y. Pyles J. A. Ward M. J. Richardson R. M. Ghuman A. S. (2016). Associative Hallucinations Result from Stimulating Left Ventromedial Temporal Cortex. Cortex 83, 139–144. 10.1016/j.cortex.2016.07.012 27533133 PMC5228589

[B3] Andrews-Hanna J. R. Reidler J. S. Sepulcre J. Poulin R. Buckner R. L. (2010). Functional-anatomic Fractionation of the Brain's Default Network. Neuron 65 (4), 550–562. 10.1016/j.neuron.2010.02.005 20188659 PMC2848443

[B4] Apud Peláez I. E. (2020). Personality Traits in Former Spanish Substance Users Recovered with Ayahuasca. J. Psychoactive Drugs 52 (3), 264–272. 10.1080/02791072.2020.1752960 32362241

[B5] Barone W. Beck J. Mitsunaga-Whitten M. Perl P. (2019). Perceived Benefits of MDMA-Assisted Psychotherapy beyond Symptom Reduction: Qualitative Follow-Up Study of a Clinical Trial for Individuals with Treatment-Resistant PTSD. J. Psychoactive Drugs 51 (2), 199–208. 10.1080/02791072.2019.1580805 30849288

[B6] Barrett F. S. Doss M. K. Sepeda N. D. Pekar J. J. Griffiths R. R. (2020). Emotions and Brain Function Are Altered up to One Month after a Single High Dose of Psilocybin. Sci. Rep. 10 (1), 2214. 10.1038/s41598-020-59282-y 32042038 PMC7010702

[B7] Barrett F. S. Krimmel S. R. Griffiths R. R. Seminowicz D. A. Mathur B. N. (2020b). Psilocybin Acutely Alters the Functional Connectivity of the Claustrum with Brain Networks that Support Perception, Memory, and Attention. NeuroImage 218, 116980. 10.1016/j.neuroimage.2020.116980 32454209 PMC10792549

[B8] Beliveau V. Ganz M. Feng L. Ozenne B. Højgaard L. Fisher P. M. (2017). A High-Resolution *In Vivo* Atlas of the Human Brain's Serotonin System. J. Neurosci. 37 (1), 120–128. 10.1523/JNEUROSCI.2830-16.2016 28053035 PMC5214625

[B9] Belser A. B. Agin-Liebes G. Swift T. C. Terrana S. Devenot N. Friedman H. L. (2017). Patient Experiences of Psilocybin-Assisted Psychotherapy: An Interpretative Phenomenological Analysis. J. Humanist. Psychol. 57 (4), 354–388. 10.1177/0022167817706884

[B10] Blanke O. Landis T. Seeck M. (2000). Electrical Cortical Stimulation of the Human Prefrontal Cortex Evokes Complex Visual Hallucinations. Epilepsy Behav. 1 (5), 356–361. 10.1006/ebeh.2000.0109 12609167

[B11] Bogenschutz M. P. Forcehimes A. A. Pommy J. A. Wilcox C. E. Barbosa P. C. Strassman R. J. (2015). Psilocybin-assisted Treatment for Alcohol Dependence: A Proof-Of-Concept Study. J. Psychopharmacol. 29 (3), 289–299. 10.1177/0269881114565144 25586396

[B12] Brohawn K. H. Offringa R. Pfaff D. L. Hughes K. C. Shin L. M. (2010). The Neural Correlates of Emotional Memory in Posttraumatic Stress Disorder. Biol. Psychiatry 68 (11), 1023–1030. 10.1016/j.biopsych.2010.07.018 20855060

[B13] Broyd S. J. Demanuele C. Debener S. Helps S. K. James C. J. Sonuga-Barke E. J. (2009). Default-mode Brain Dysfunction in Mental Disorders: A Systematic Review. Neurosci. Biobehav Rev. 33 (3), 279–296. 10.1016/j.neubiorev.2008.09.002 18824195

[B14] Buckner R. L. Andrews-Hanna J. R. Schacter D. L. (2008). The Brain's Default Network. Ann. N. Y Acad. Sci. 1124, 1–38. 10.1196/annals.1440.011 18400922

[B15] Carhart-Harris R. L. Erritzoe D. Williams T. Stone J. M. Reed L. J. Colasanti A. (2012). Neural Correlates of the Psychedelic State as Determined by fMRI Studies with Psilocybin. Proc. Natl. Acad. Sci. U S A. 109 (6), 2138–2143. 10.1073/pnas.1119598109 22308440 PMC3277566

[B16] Carhart-Harris R. L. Bolstridge M. Rucker J. Day C. M. Erritzoe D. Kaelen M. (2016a). Psilocybin with Psychological Support for Treatment-Resistant Depression: an Open-Label Feasibility Study. Lancet Psychiatry 3 (7), 619–627. 10.1016/S2215-0366(16)30065-7 27210031

[B17] Carhart-Harris R. L. Muthukumaraswamy S. Roseman L. Kaelen M. Droog W. Murphy K. (2016b). Neural Correlates of the LSD Experience Revealed by Multimodal Neuroimaging. Proc. Natl. Acad. Sci. U S A. 113 (17), 4853–4858. 10.1073/pnas.1518377113 27071089 PMC4855588

[B18] Carhart-Harris R. L. Kaelen M. Bolstridge M. Williams T. M. Williams L. T. Underwood R. (2016c). The Paradoxical Psychological Effects of Lysergic Acid Diethylamide (LSD). Psychol. Med. 46 (7), 1379–1390. 10.1017/S0033291715002901 26847689

[B19] Carhart-Harris R. L. Roseman L. Bolstridge M. Demetriou L. Pannekoek J. N. Wall M. B. (2017). Psilocybin for Treatment-Resistant Depression: FMRI-Measured Brain Mechanisms. Sci. Rep. 7 (1), 13187. 10.1038/s41598-017-13282-7 29030624 PMC5640601

[B20] Castellanos J. P. Woolley C. Bruno K. A. Zeidan F. Halberstadt A. Furnish T. (2020). Chronic Pain and Psychedelics: A Review and Proposed Mechanism of Action. Reg. Anesth. Pain Med. 45 (7), 486–494. 10.1136/rapm-2020-101273 32371500

[B21] Császár-Nagy N. Kapócs G. Bókkon I. (2019). Classic Psychedelics: The Special Role of the Visual System. Rev. Neurosci. 30 (6), 651–669. 10.1515/revneuro-2018-0092 30939118

[B22] Cumming P. Scheidegger M. Dornbierer D. Palner M. Quednow B. B. Martin-Soelch C. (2021). Molecular and Functional Imaging Studies of Psychedelic Drug Action in Animals and Humans. Molecules 26 (9), 2451. 10.3390/molecules26092451 33922330 PMC8122807

[B23] Danforth A. L. Grob C. S. Struble C. Feduccia A. A. Walker N. Jerome L. (2018). Reduction in Social Anxiety after MDMA-Assisted Psychotherapy with Autistic Adults: a Randomized, Double-Blind, Placebo-Controlled Pilot Study. Psychopharmacology (Berl) 235 (11), 3137–3148. 10.1007/s00213-018-5010-9 30196397 PMC6208958

[B24] Daumann J. Wagner D. Heekeren K. Neukirch A. Thiel C. M. Gouzoulis-Mayfrank E. (2010). Neuronal Correlates of Visual and Auditory Alertness in the DMT and Ketamine Model of Psychosis. J. Psychopharmacol. 24 (10), 1515–1524. 10.1177/0269881109103227 19304859

[B25] De Araujo D. B. Ribeiro S. Cecchi G. A. Carvalho F. M. Sanchez T. A. Pinto J. P. (2012). Seeing with the Eyes Shut: Neural Basis of Enhanced Imagery Following Ayahuasca Ingestion. Hum. Brain Mapp. 33 (11), 2550–2560. 10.1002/hbm.21381 21922603 PMC6870240

[B26] de Gregorio D. Popic J. Enns J. P. Inserra A. Skalecka A. Markopoulos A. (2021). Lysergic Acid Diethylamide (LSD) Promotes Social Behavior through mTORC1 in the Excitatory Neurotransmission. Proc. Natl. Acad. Sci. U S A. 118 (5), e2020705118. 10.1073/pnas.2020705118 33495318 PMC7865169

[B27] Díaz J. L. (2010). Sacred Plants and Visionary Consciousness. Phenom Cogn. Sci. 9 (2), 159–170. 10.1007/s11097-010-9157-z

[B28] Dolder P. C. Schmid Y. Müller F. Borgwardt S. Liechti M. E. (2016). LSD Acutely Impairs Fear Recognition and Enhances Emotional Empathy and Sociality. Neuropsychopharmacology 41 (11), 2638–2646. 10.1038/npp.2016.82 27249781 PMC5026740

[B29] Domínguez-Clavé E. Soler J. Elices M. Pascual J. C. Álvarez E. de la Fuente Revenga M. (2016). Ayahuasca: Pharmacology, Neuroscience and Therapeutic Potential. Brain Res. Bull. 126, 89–101. 10.1016/j.brainresbull.2016.03.002 26976063

[B30] dos Santos R. G. Osório F. L. Crippa J. A. S. Hallak J. E. C. (2016). Classical Hallucinogens and Neuroimaging: A Systematic Review of Human Studies. Neurosci. Biobehav. Rev. 71, 715–728. 10.1016/j.neubiorev.2016.10.026 27810345

[B31] Duncan S. Barrett L. F. (2007). The Role of the Amygdala in Visual Awareness. Trends Cogn. Sci. 11 (5), 190–192. 10.1016/j.tics.2007.01.007 17360224 PMC2234439

[B32] Eickhoff S. B. Nichols T. E. Laird A. R. Hoffstaedter F. Amunts K. Fox P. T. (2016). Behavior, Sensitivity, and Power of Activation Likelihood Estimation Characterized by Massive Empirical Simulation. Neuroimage 137, 70–85. 10.1016/j.neuroimage.2016.04.072 27179606 PMC4981641

[B33] Etkin A. Wager T. D. (2007). Functional Neuroimaging of Anxiety: a Meta-Analysis of Emotional Processing in PTSD, Social Anxiety Disorder, and Specific Phobia. Am. J. Psychiatry 164 (10), 1476–1488. 10.1176/appi.ajp.2007.07030504 17898336 PMC3318959

[B34] Freese J. L. Amaral D. G. (2005). The Organization of Projections from the Amygdala to Visual Cortical Areas TE and V1 in the Macaque Monkey. J. Comp. Neurol. 486 (4), 295–317. 10.1002/cne.20520 15846786

[B35] Furl N. Henson R. N. Friston K. J. Calder A. J. (2013). Top-down Control of Visual Responses to Fear by the Amygdala. J. Neurosci. 33 (44), 17435–17443. 10.1523/JNEUROSCI.2992-13.2013 24174677 PMC6618361

[B36] Garrity A. G. Pearlson G. D. McKiernan K. Lloyd D. Kiehl K. A. Calhoun V. D. (2007). Aberrant “Default Mode” Functional Connectivity in Schizophrenia. Am. J. Psychiatry 164 (3), 450–457. 10.1176/ajp.2007.164.3.450 17329470

[B37] Gasser P. Holstein D. Michel Y. Doblin R. Yazar-Klosinski B. Passie T. (2014). Safety and Efficacy of Lysergic Acid Diethylamide-Assisted Psychotherapy for Anxiety Associated with Life-Threatening Diseases. J. Nerv Ment. Dis. 202 (7), 513–520. 10.1097/NMD.0000000000000113 24594678 PMC4086777

[B38] Gasser P. Kirchner K. Passie T. (2015). LSD-assisted Psychotherapy for Anxiety Associated with a Life-Threatening Disease: A Qualitative Study of Acute and Sustained Subjective Effects. J. Psychopharmacol. 29 (1), 57–68. 10.1177/0269881114555249 25389218

[B39] Gouzoulis-Mayfrank E. Schreckenberger M. Sabri O. Arning C. Thelen B. Spitzer M. (1999). Neurometabolic Effects of Psilocybin, 3,4-methylenedioxyethylamphetamine (MDE) and D-Methamphetamine in Healthy Volunteers. A Double-Blind, Placebo-Controlled PET Study with [18F]FDG. Neuropsychopharmacology 20 (6), 565–581. 10.1016/S0893-133X(98)00089-X 10327426

[B40] Griffiths R. R. Johnson M. W. Carducci M. A. Umbricht A. Richards W. A. Richards B. D. (2016). Psilocybin Produces Substantial and Sustained Decreases in Depression and Anxiety in Patients with Life-Threatening Cancer: A Randomized Double-Blind Trial. J. Psychopharmacol. 30 (12), 1181–1197. 10.1177/0269881116675513 27909165 PMC5367557

[B41] Halberstadt A. L. Geyer M. A. (2011). Multiple Receptors Contribute to the Behavioral Effects of Indoleamine Hallucinogens. Neuropharmacology 61 (3), 364–381. 10.1016/j.neuropharm.2011.01.017 21256140 PMC3110631

[B42] Holzschneider K. Mulert C. (2011). Neuroimaging in Anxiety Disorders. Dialogues Clin. Neurosci. 13 (4), 453–461. 10.31887/dcns.2011.13.4/kholzschneider 22275850 PMC3263392

[B43] Johnson M. W. Garcia-Romeu A. Cosimano M. P. Griffiths R. R. (2014). Pilot Study of the 5-HT2AR Agonist Psilocybin in the Treatment of Tobacco Addiction. J. Psychopharmacol. 28 (11), 983–992. 10.1177/0269881114548296 25213996 PMC4286320

[B44] Kaelen M. Roseman L. Kahan J. Santos-Ribeiro A. Orban C. Lorenz R. (2016). LSD Modulates Music-Induced Imagery via Changes in Parahippocampal Connectivity. Eur. Neuropsychopharmacol. 26 (7), 1099–1109. 10.1016/j.euroneuro.2016.03.018 27084302

[B45] Kapur S. (2003). Psychosis as a State of Aberrant Salience: A Framework Linking Biology, Phenomenology, and Pharmacology in Schizophrenia. Am. J. Psychiatry 160 (1), 13–23. 10.1176/appi.ajp.160.1.13 12505794

[B46] Klüver H. (1942). “Mechanisms of Hallucinations,” in Studies in Personality. Editors McNemar Q. Merrill M. A. (New York: McGraw-Hill), 175–207.

[B47] Kochunov P. Lancaster J. Thompson P. Toga A. W. Brewer P. Hardies J. (2002). An Optimized Individual Target Brain in the Talairach Coordinate System. Neuroimage 17, 922–927. 10.1006/nimg.2002.1084 12377166

[B48] Kometer M. Vollenweider F. X. (2018). Serotonergic Hallucinogen-Induced Visual Perceptual Alterations. Curr. Top. Behav. Neurosci. 36, 257–282. 10.1007/7854_2016_461 27900674

[B49] Kometer M. Schmidt A. Jäncke L. Vollenweider F. X. (2013). Activation of Serotonin 2A Receptors Underlies the Psilocybin-Induced Effects on α Oscillations, N170 Visual-Evoked Potentials, and Visual Hallucinations. J. Neurosci. 33 (25), 10544–10551. 10.1523/JNEUROSCI.3007-12.2013 23785166 PMC6618596

[B50] Kometer M. Pokorny T. Seifritz E. Volleinweider F. X. (2015). Psilocybin-induced Spiritual Experiences and Insightfulness Are Associated with Synchronization of Neuronal Oscillations. Psychopharmacology (Berl) 232 (19), 3663–3676. 10.1007/s00213-015-4026-7 26231498

[B51] Kraehenmann R. Preller K. H. Scheidegger M. Pokorny T. Bosch O. G. Seifritz E. (2015). Psilocybin-induced Decrease in Amygdala Reactivity Correlates with Enhanced Positive Mood in Healthy Volunteers. Biol. Psychiatry 78 (8), 572–581. 10.1016/j.biopsych.2014.04.010 24882567

[B52] Kraehenmann R. Schmidt A. Friston K. Preller K. H. Seifritz E. Vollenweider F. X. (2016). The Mixed Serotonin Receptor Agonist Psilocybin Reduces Threat-Induced Modulation of Amygdala Connectivity. Neuroimage Clin. 11, 53–60. 10.1016/j.nicl.2015.08.009 26909323 PMC4732191

[B53] Kraehenmann R. (2017). Dreams and Psychedelics: Neurophenomenological Comparison and Therapeutic Implications. Curr. Neuropharmacol. 15, 1032–1042. 10.2174/1573413713666170619092629 28625125 PMC5652011

[B54] Li W. Mai X. Liu C. (2014). The Default Mode Network and Social Understanding of Others: what Do Brain Connectivity Studies Tell Us. Front. Hum. Neurosci. 8, 74. 10.3389/fnhum.2014.0007410.3389/fnhum.2014.00074 24605094 PMC3932552

[B55] Lowe H. Toyang N. Steele B. Valentine H. Grant J. Ali A. (2021). The Therapeutic Potential of Psilocybin. Molecules 26 (10), 2948. 10.3390/molecules26102948 34063505 PMC8156539

[B56] Luppi A. I. Carhart-Harris R. L. Roseman L. Pappas I. Menon D. K. Stamatakis E. A. (2021). LSD Alters Dynamic Integration and Segregation in the Human Brain. NeuroImage 227, 117653. 10.1016/j.neuroimage.2020.117653 33338615 PMC7896102

[B57] Madsen M. K. Stenbæk D. S. Armand S. Marstrand-Joergensen M. R. Johansen S. S. Linnet K. (2021). Psilocybin-induced Changes in Brain Network Integrity and Segregation Correlate with Plasma Psilocin Level and Psychedelic Experience. Eur. Neuropsychopharmacol. 50, 121–132. 10.1016/j.euroneuro.2021.06.001 34246868

[B58] Mars R. B. Neubert F. X. Noonan M. P. Sallet J. Toni I. Rushworth M. F. (2012). On the Relationship between the "default Mode Network" and the "social Brain". Front. Hum. Neurosci. 6, 189. 10.3389/fnhum.2012.00189 22737119 PMC3380415

[B59] Mason N. L. Mischler E. Uthaug M. V. Kuypers K. P. C. (2019). Sub-Acute Effects of Psilocybin on Empathy, Creative Thinking, and Subjective Well-Being. J. Psychoactive Drugs 51 (2), 123–134. 10.1080/02791072.2019.1580804 30905276

[B60] Mason N. L. Kuypers K. P. C. Reckweg J. T. Müller F. Tse D. H. Y. Da Rios B. (2021). Spontaneous and Deliberate Creative Cognition during and after Psilocybin Exposure. Transl Psychiatry 11, 209. 10.1038/s41398-021-01335-5 33833225 PMC8032715

[B61] McKenna D. Riba J. (2018). New World Tryptamine Hallucinogens and the Neuroscience of Ayahuasca. Curr. Top. Behav. Neurosci. 36, 283–311. 10.1007/7854_2016_472 28401525

[B62] Mégevand P. Groppe D. M. Goldfinger M. S. Hwang S. T. Kingsley P. B. Davidesco I. (2014). Seeing Scenes: Topographic Visual Hallucinations Evoked by Direct Electrical Stimulation of the Parahippocampal Place Area. J. Neurosci. 34 (16), 5399–5405. 10.1523/JNEUROSCI.5202-13.2014 24741031 PMC6608225

[B63] Menon V. (2015). “Salience Network,” in Brain Mapping: An Encyclopedic Reference (New York: Elsevier), 2, 597–611. 10.1016/B978-0-12-397025-1.00052-X

[B64] Mitchell J. M. Bogenschutz M. Lilienstein A. Harrison C. Kleiman S. Parker-Guilbert K. (2021). MDMA-assisted Therapy for Severe PTSD: a Randomized, Double-Blind, Placebo-Controlled Phase 3 Study. Nat. Med. 27, 1025–1033. 10.1038/s41591-021-01336-3 33972795 PMC8205851

[B65] Moreau A. W. Amar M. Le Roux N. Morel N. Fossier P. (2010). Serotoninergic fine-tuning of the Excitation-Inhibition Balance in Rat Visual Cortical Networks. Cereb. Cortex 20 (2), 456–467. 10.1093/cercor/bhp114 19520765

[B66] Moreno F. A. Wiegand C. B. Taitano E. K. Delgado P. L. (2006). Safety, Tolerability, and Efficacy of Psilocybin in 9 Patients with Obsessive-Compulsive Disorder. J. Clin. Psychiatry 67 (11), 1735–1740. 10.4088/JCP.v67n1110 17196053

[B67] Mueller F. Lenz C. Dolder P. C. Harder S. Schmid Y. Lang U. E. (2017). Acute Effects of LSD on Amygdala Activity during Processing of Fearful Stimuli in Healthy Subjects. Transl Psychiatry 7 (4), e1084. 10.1038/tp.2017.54 28375205 PMC5416695

[B68] Netzband N. Ruffell S. Linton S. Tsang W. F. Wolff T. (2020). Modulatory Effects of Ayahuasca on Personality Structure in a Traditional Framework. Psychopharmacology (Berl) 237 (10), 3161–3171. 10.1007/s00213-020-05601-0 32700023 PMC7524857

[B69] Nichols D. E. (2004). Hallucinogens. Pharmacol. Ther. 101 (2), 131–181. 10.1016/j.pharmthera.2003.11.002 14761703

[B70] Noorani T. Garcia-Romeu A. Swift T. C. Griffiths R. R. Johnson M. W. (2018). Psychedelic Therapy for Smoking Cessation: Qualitative Analysis of Participant Accounts. J. Psychopharmacol. 32 (7), 756–769. 10.1177/0269881118780612 29938565

[B71] Nour M. M. Evans L. Nutt D. Carhart-Harris R. L. (2016). Ego-dissolution and Psychedelics: Validation of the Ego-Dissolution Inventory (EDI). Front. Hum. Neurosci. 10, 269. 10.3389/fnhum.2016.00269 27378878 PMC4906025

[B72] Palaniyappan L. Liddle P. F. (2012). Does the Salience Network Play a Cardinal Role in Psychosis? an Emerging Hypothesis of Insular Dysfunction. J. Psychiatry Neurosci. 37 (1), 17–27. 10.1503/jpn.100176 21693094 PMC3244495

[B73] Palhano-Fontes F. Andrade K. C. Tofoli L. F. Santos A. C. Crippa J. A. Hallak J. E. (2015). The Psychedelic State Induced by Ayahuasca Modulates the Activity and Connectivity of the Default Mode Network. PLoS ONE 10 (2), e0118143. 10.1371/journal.pone.0118143 25693169 PMC4334486

[B74] Palhano-Fontes F. Barreto D. Onias H. Andrade K. C. Novaes M. M. Pessoa J. A. (2019). Rapid Antidepressant Effects of the Psychedelic Ayahuasca in Treatment-Resistant Depression: A Randomized Placebo-Controlled Trial. Psychol. Med. 49 (4), 655–663. 10.1017/S0033291718001356 29903051 PMC6378413

[B75] Pang Y. Cui Q. Wang Y. Chen Y. Wang X. Han S. (2016). Extraversion and Neuroticism Related to the Resting-State Effective Connectivity of Amygdala. Sci. Rep. 6, 35484. 10.1038/srep35484 27765947 PMC5073227

[B76] Petri G. Expert P. Turkheimer F. Carhart-Harris R. Nutt D. Hellyer P. J. (2014). Homological Scaffolds of Brain Functional Networks. J. R. Soc. Interf. 11 (101), 20140873. 10.1098/rsif.2014.0873 PMC4223908 25401177

[B77] Pokorny T. Preller K. H. Kometer M. Dziobek I. Vollenweider F. X. (2017). Effect of Psilocybin on Empathy and Moral Decision-Making. Int. J. Neuropsychopharmacol. 20 (9), 747–757. 10.1093/ijnp/pyx047 28637246 PMC5581487

[B78] Preller K. H. Vollenweider F. X. (2019). Modulation of Social Cognition via Hallucinogens and "Entactogens". Front. Psychiatry 10, 881. 10.3389/fpsyt.2019.00881 31849730 PMC6902301

[B79] Preller K. H. Pokorny T. Hock A. Kraehenmann R. Stämpfli P. Seifritz E. (2016). Effects of Serotonin 2A/1A Receptor Stimulation on Social Exclusion Processing. Proc. Natl. Acad. Sci. U S A. 113 (18), 5119–5124. 10.1073/pnas.1524187113 27091970 PMC4983864

[B80] Preller K. H. Herdener M. Pokorny T. Planzer A. Kraehenmann R. Stämpfli P. (2017). The Fabric of Meaning and Subjective Effects in LSD-Induced States Depend on Serotonin 2A Receptor Activation. Curr. Biol. 27 (3), 451–457. 10.1016/j.cub.2016.12.030 28132813

[B81] Preller K. H. Schilbach L. Pokorny T. Flemming J. Seifritz E. Vollenweider F. X. (2018). Role of the 5-HT2A Receptor in Self- and Other-Initiated Social Interaction in Lysergic Acid Diethylamide-Induced States: A Pharmacological fMRI Study. J. Neurosci. 38, 3603–3611. 10.1523/JNEUROSCI.1939-17.2018 29555857 PMC6596041

[B82] Quednow B. B. Kometer M. Geyer M. A. Vollenweider F. X. (2012). Psilocybin-induced Deficits in Automatic and Controlled Inhibition Are Attenuated by Ketanserin in Healthy Human Volunteers. Neuropsychopharmacology 37 (3), 630–640. 10.1038/npp.2011.228 21956447 PMC3260978

[B83] Riba J. Anderer P. Jané F. Saletu B. Barbanoj M. J. (2004). Effects of the South American Psychoactive Beverage Ayahuasca on Regional Brain Electrical Activity in Humans: A Functional Neuroimaging Study Using Low-Resolution Electromagnetic Tomography. Neuropsychobiology 50 (1), 89–101. 10.1159/000077946 15179026

[B84] Riba J. Romero S. Grasa E. Mena E. Carrió I. Barbanoj M. J. (2006). Increased Frontal and Paralimbic Activation Following Ayahuasca, the Pan-Amazonian Inebriant. Psychopharmacology (Berl) 186 (1), 93–98. 10.1007/s00213-006-0358-7 16575552

[B85] Rocha J. M. Osório F. L. Crippa J. A. S. Bouso J. C. Rossi G. N. Hallak J. E. C. (2019). Serotonergic Hallucinogens and Recognition of Facial Emotion Expressions: a Systematic Review of the Literature. Ther. Adv. Psychopharmacol. 9, 2045125319845774. 10.1177/2045125319845774 31065350 PMC6487767

[B86] Roseman L. Demetriou L. Wall M. B. Nutt D. J. Carhart-Harris R. L. (2018). Increased Amygdala Responses to Emotional Faces after Psilocybin for Treatment-Resistant Depression. Neuropharmacology 142, 263–269. 10.1016/j.neuropharm.2017.12.041 29288686

[B87] Ross S. Bossis A. Guss J. Agin-Liebes G. Malone T. Cohen B. (2016). Rapid and Sustained Symptom Reduction Following Psilocybin Treatment for Anxiety and Depression in Patients with Life-Threatening Cancer: A Randomized Controlled Trial. J. Psychopharmacol. 30 (12), 1165–1180. 10.1177/0269881116675512 27909164 PMC5367551

[B88] Rummele W. Gnirss F. (1961). Untersuchungen mit Psilocybin, einer Psychotropen Substanz aus Psilocybe mexicana. [Studies with Psilocybin, a Psychotropic Substance from Psilocybe mexicana. Chweizer Archiv Fur Neurologie Und Psychiatrie 87 (2), 365–385. 13744546

[B89] Schmid Y. Enzler F. Gasser P. Grouzmann E. Preller K. H. Vollenweider F. X. (2015). Acute Effects of Lysergic Acid Diethylamide in Healthy Subjects. Biol. Psychiatry 78 (8), 544–553. 10.1016/j.biopsych.2014.11.015 25575620

[B90] Schmidt A. Müller F. Lenz C. Dolder P. C. Schmid Y. Zanchi D. (2018). Acute LSD Effects on Response Inhibition Neural Networks. Psychol. Med. 48 (9), 1464–1473. 10.1017/S0033291717002914 28967351

[B91] Seillier L. Lorenz C. Kawaguchi K. Ott T. Nieder A. Pourriahi P. (2017). Serotonin Decreases the Gain of Visual Responses in Awake Macaque V1. J. Neurosci. 37 (47), 11390–11405. 10.1523/JNEUROSCI.1339-17.2017 29042433 PMC5700422

[B92] Shanon B. (2010). The Epistemics of Ayahuasca Visions. Phenom Cogn. Sci. 9 (2), 263–280. 10.1007/s11097-010-9161-3

[B93] Sheline Y. I. Barch D. M. Price J. L. Rundle M. M. Vaishnavi S. N. Snyder A. Z. (2009). The Default Mode Network and Self-Referential Processes in Depression. Proc. Natl. Acad. Sci. U S A. 106 (6), 1942–1947. 10.1073/pnas.0812686106 19171889 PMC2631078

[B94] Silani G. Lamm C. Ruff C. C. Singer T. (2013). Right Supramarginal Gyrus Is Crucial to Overcome Emotional Egocentricity Bias in Social Judgments. J. Neurosci. 33 (39), 15466–15476. 10.1523/JNEUROSCI.1488-13.2013 24068815 PMC6618458

[B95] Stopyra M. A. Simon J. J. Skunde M. Walther S. Bendszus M. Herzog W. (2019). Altered Functional Connectivity in Binge Eating Disorder and Bulimia Nervosa: A Resting-State fMRI Study. Brain Behav. 9 (2), e01207. 10.1002/brb3.1207 30644179 PMC6379643

[B96] Švob Štrac D. Pivac N. Mück-Šeler D. (2016). The Serotonergic System and Cognitive Function. Transl. Neurosci. 7 (1), 35–49. 10.1515/tnsci-2016-0007 28123820 PMC5017596

[B97] Studerus E. Kometer M. Hasler F. Vollenweider F. X. (2011). Acute, Subacute and Long-Term Subjective Effects of Psilocybin in Healthy Humans: A Pooled Analysis of Experimental Studies. J. Psychopharmacol. 25 (11), 1434–1452. 10.1177/0269881110382466 20855349

[B98] Swanson L. R. (2018). Unifying Theories of Psychedelic Drug Effects. Front. Pharmacol. 9, 172. 10.3389/fphar.2018.00172 29568270 PMC5853825

[B99] Swift T. C. Belser A. B. Agin-Liebes G. Devenot N. Terrana S. Friedman H. L. (2017). Cancer at the Dinner Table: Experiences of Psilocybin-Assisted Psychotherapy for the Treatment of Cancer-Related Distress. J. Humanist. Psychol. 57 (5), 488–519. 10.1177/0022167817715966

[B100] Tagliazucchi E. Carhart-Harris R. Leech R. Nutt D. Chialvo D. R. (2014). Enhanced Repertoire of Brain Dynamical States during the Psychedelic Experience. Hum. Brain Mapp. 35 (11), 5442–5456. 10.1002/hbm.22562 24989126 PMC6869695

[B101] Tagliazucchi E. Roseman L. Kaelen M. Orban C. Muthukumaraswamy S. D. Murphy K. (2016). Increased Global Functional Connectivity Correlates with LSD-Induced Ego Dissolution. Curr. Biol. 26 (8), 1043–1050. 10.1016/j.cub.2016.02.010 27085214

[B102] Teixeira P. J. Johnson M. W. Timmermann C. Watts R. Erritzoe D. Douglass H. (2021). Psychedelics and Health Behaviour Change. J. Psychopharmacol., 026988112110085. 10.1177/02698811211008554 PMC8801670 34053342

[B103] Thorsen A. L. Hagland P. Radua J. Mataix-Cols D. Kvale G. Hansen B. (2018). Emotional Processing in Obsessive-Compulsive Disorder: A Systematic Review and Meta-Analysis of 25 Functional Neuroimaging Studies. Biol. Psychiatry Cogn. Neurosci. Neuroimaging 3 (6), 563–571. 10.1016/j.bpsc.2018.01.009 29550459 PMC5994188

[B104] Turkeltaub P. E. Eden G. F. Jones K. M. Zeffiro T. A. (2002). Meta-analysis of the Functional Neuroanatomy of Single-word reading: Method and Validation. Neuroimage 16, 765–780. 10.1006/nimg.2002.1131 12169260

[B105] Uddin L. Q. (2015). Salience Processing and Insular Cortical Function and Dysfunction. Nat. Rev. Neurosci. 16 (1), 55–61. 10.1038/nrn3857 25406711

[B106] Uthaug M. V. Mason N. L. Toennes S. W. Reckweg J. T. de Sousa Fernandes Perna E. B. Kuypers K. P. C. (2021). A Placebo-Controlled Study of the Effects of Ayahuasca, Set and Setting on Mental Health of Participants in Ayahuasca Group Retreats. Psychopharmacology (Berl) 238, 1899–1910. 10.1007/s00213-021-05817-8 33694031 PMC8233273

[B107] Van Eimeren T. Monchi O. Ballanger B. Strafella A. P. (2009). Dysfunction of the Default Mode Network in Parkinson Disease: A Functional Magnetic Resonance Imaging Study. Arch. Neurol. 66 (7), 877–883. 10.1001/archneurol.2009.97 19597090 PMC2972248

[B108] Vann Jones S. A. O'Kelly A. (2020). Psychedelics as a Treatment for Alzheimer's Disease Dementia. Front. Synaptic Neurosci. 12, 34. 10.3389/fnsyn.2020.00034 32973482 PMC7472664

[B109] Vollenweider F. X. Kometer M. (2010). The Neurobiology of Psychedelic Drugs: Implications for the Treatment of Mood Disorders. Nat. Rev. Neurosci. 11 (9), 642–651. 10.1038/nrn2884 20717121

[B110] Vollenweider F. X. Leenders K. L. Scharfetter C. Maguire P. Stadelmann O. Angst J. (1997). Positron Emission Tomography and Fluorodeoxyglucose Studies of Metabolic Hyperfrontality and Psychopathology in the Psilocybin Model of Psychosis. Neuropsychopharmacology 16 (5), 357–372. 10.1016/S0893-133X(96)00246-1 9109107

[B111] Vollenweider F. X. Vontobel P. Hell D. Leenders K. L. (1999). 5-HT Modulation of Dopamine Release in Basal Ganglia in Psilocybin-Induced Psychosis in Man-Aa PET Study with [11C]raclopride. Neuropsychopharmacology 20 (5), 424–433. 10.1016/S0893-133X(98)00108-0 10192823

[B112] Vuilleumier P. Richardson M. P. Armony J. L. Driver J. Dolan R. J. (2004). Distant Influences of Amygdala Lesion on Visual Cortical Activation during Emotional Face Processing. Nat. Neurosci. 7 (11), 1271–1278. 10.1038/nn1341 15494727

[B113] Watts R. Day C. Krzanowski J. Nutt D. Carhart-Harris R. (2017). Patients' Accounts of Increased "Connectedness" and "Acceptance" after Psilocybin for Treatment-Resistant Depression. J. Humanist. Psychol. 57 (5), 520–564. 10.1177/0022167817709585

[B114] Zhang R. Volkow N. D. (2019). Brain Default-Mode Network Dysfunction in Addiction. NeuroImage 200, 313–331. 10.1016/j.neuroimage.2019.06.036 31229660

